# Geographical Variation in Adrenocortical Carcinoma Incidence Across Colorado

**DOI:** 10.1210/jendso/bvaf057

**Published:** 2025-04-08

**Authors:** Tessa B Holmstoen, Lucy K Volino, Elizabeth Molina Kuna, Tapahsama Banerjee, Lauren Fishbein, Margaret E Wierman, Katja Kiseljak-Vassiliades

**Affiliations:** Division of Endocrinology, Metabolism and Diabetes, Department of Medicine, University of Colorado Anschutz Medical Campus, Aurora, CO 80045, USA; Research Service, Rocky Mountain Regional Veterans Affairs Medical Center, Aurora, CO 80045, USA; Division of Endocrinology, Metabolism and Diabetes, Department of Medicine, University of Colorado Anschutz Medical Campus, Aurora, CO 80045, USA; Cancer Center Population Health Shared Resource, University of Colorado Anschutz Medical Campus, Aurora, CO 80045, USA; Division of Endocrinology, Metabolism and Diabetes, Department of Medicine, University of Colorado Anschutz Medical Campus, Aurora, CO 80045, USA; Research Service, Rocky Mountain Regional Veterans Affairs Medical Center, Aurora, CO 80045, USA; Division of Endocrinology, Metabolism and Diabetes, Department of Medicine, University of Colorado Anschutz Medical Campus, Aurora, CO 80045, USA; Research Service, Rocky Mountain Regional Veterans Affairs Medical Center, Aurora, CO 80045, USA; Division of Endocrinology, Metabolism and Diabetes, Department of Medicine, University of Colorado Anschutz Medical Campus, Aurora, CO 80045, USA; Research Service, Rocky Mountain Regional Veterans Affairs Medical Center, Aurora, CO 80045, USA; Division of Endocrinology, Metabolism and Diabetes, Department of Medicine, University of Colorado Anschutz Medical Campus, Aurora, CO 80045, USA; Research Service, Rocky Mountain Regional Veterans Affairs Medical Center, Aurora, CO 80045, USA

**Keywords:** adrenocortical carcinoma, pheochromocytoma/paraganglioma, epidemiology

## Abstract

Adrenocortical carcinoma (ACC) is an aggressive endocrine malignancy with an annual incidence of approximately 1 case per million, with the underlying etiology poorly understood. We retrospectively investigated the geographic distribution of 62 ACC cases diagnosed between 2010 and 2023 and of 115 pheochromocytoma/paraganglioma (PPGL) diagnosed between 2016 and 2023 at the University of Colorado Hospital, as well as 115 ACC cases diagnosed between 2012 and 2020 from the Colorado Central Cancer Registry (CCCR). Data on patient age, sex, zip code of residence, and tumor characteristics were collected and, for ACC cases, compared with CCCR data. Our University of Colorado cohort showed an average ACC annual incidence of 0.81 cases per million, with 61.2% of cases occurring in women. The CCCR cohort showed an average ACC annual incidence of 1.1 cases per million, with 48.7% of cases in women. For PPGL, the average annual incidence was 2.26 cases per million, with 60% of cases occurring in females. Our ACC cohort had an average annual incidence of 1.36 cases per million in Western Colorado and 0.68 cases per million in Eastern Colorado. Similarly, the state registry showed 1.49 cases per million in Western Colorado and 1 case per million in Eastern Colorado. In contrast, PPGL data showed 1.35 cases per million in Western Colorado and 2.36 cases per million in Eastern Colorado. These data suggest a higher incidence of ACC in Western Colorado, highlighting the need for investigation into environmental factors as potential pathogenic factors in ACC.

Adrenocortical carcinoma (ACC) is a rare and potentially deadly endocrine malignancy, with an annual incidence of around 1 case per million [1, 2]. Around 60% of cases have a known germline or somatic genetic driver [3], but 40% of cases have no known driver. The potential environmental contributors are largely unexplained to date. Recently, Balderrama-Brondani et al [4] examined the geographic distribution of 448 ACC cases in the Texas State Cancer Registry, but did not find a region-specific distribution. Additionally, Puglisi et al [5] reported that residents of areas in Italy with contaminated soil and water had a statistically smaller incidence of ACC; however, ACC patients residing in these areas had a shorter disease-free survival than those in noncontaminated areas. We hypothesized that there may be a differential rate of presentation of ACC patients in our Adrenal Tumor Multidisciplinary Program at the University of Colorado (CU) dependent on location across our state. To make an objective comparison, we compared the geographic location of patients presenting with ACC to those with aciher type of adrenal tumor, pheochromocytoma/paraganglioma (PPGL).

## Materials and Methods

**Figure 1.**
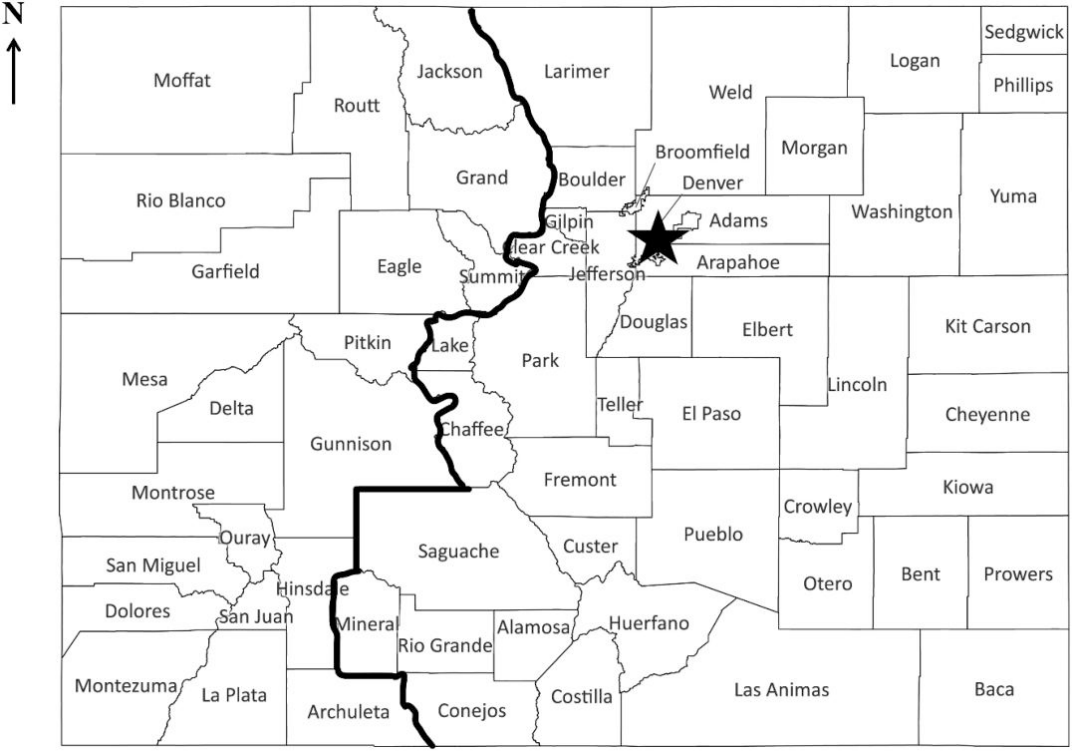
The designation of Western and Eastern Colorado regions, with the star indicating the location of our center at the University of Colorado Anschutz Medical Center.

This population-based, retrospective cohort study, approved by the Colorado Institutional Review Board, included analysis of data from 62 consecutive ACC patients evaluated in our program at least once, and diagnosed with ACC between 2010 and 2023. Data collection included each patient's age at diagnosis, date of diagnosis, sex, zip code of residence, tumor location(s), and tumor hormone secretion at diagnosis. Using the Colorado Central Cancer Registry (CCCR) data, age, sex, and zip codes of residence were identified for patients diagnosed with ACC anywhere in the state between 2012 and 2020. The population data were collected from the 2020 Colorado census, and age-adjustment was performed using the US 2000 Standard Population obtained from the Surveillance, Epidemiology, and End Results program. To determine whether each ACC patient resided in Western or Eastern Colorado, patient zip codes were grouped into their respective counties. There are 21 counties considered to be in Western Colorado by the state demography office, and the remaining 43 counties were considered to be in Eastern Colorado for this analysis (Fig. 1).

For a relevant comparison group, similar information was collected from 115 PPGL patients seen at our center who were diagnosed for the first time with PPGL between 2016 and 2023. No CCCR data were available for PPGL. Analyses were performed in SAS 9.4 (SAS Institute Inc) as well as Python accessed via Jupyter Notebook, using libraries including NumPy and Pandas.

## Results

Sixty-two incident consented cases of ACC from Colorado were managed in our adult Adrenal Cancer Center Program between 2010 and 2023. Given that the state population is 5.7 million, this equates to an age-adjusted average annual incidence of 0.76 cases per million. Out of the 62 patient cases, 38 (61.3%) were female and 24 (38.7%) were male. The average age at diagnosis was 48.9 years, with patient and tumor characteristics shown in Table 1. Hormonal hypersecretion was apparent in 40 (54.8%) ACC tumors, whereas 22 (35.5%) tumors were nonproductive. Out of the 40 productive tumors, 34 produced cortisol, 4 produced aldosterone, 11 produced testosterone, 3 produced estradiol, and 7 produced androstenedione (see Table 1).

**Table 1.** Demographic and clinical variables of 62 adrenocortical carcinoma (ACC) patients in our University of Colorado cohort, and 115 ACC patients in the Colorado State Cancer Registry Cohort

	CU ACC cohort			CCCR ACC cohort		
Variable	Western CO patients	Eastern CO patients	Total CO patients	Western CO patients	Eastern CO patients	Total CO patients
	N (%)	N (%)	N (%)	N (%)	N (%)	N (%)
**Sex**						
Female	7 (63.63)	31 (60.78)	38 (61.20)	13 (50.00)	46 (51.69)	59 (51.30)
Male	4 (36.37)	20 (39.22)	24 (38.70)	13 (50.00)	43 (48.31)	56 (48.70)
**Age, years**						
<20	0 (0.00)	1 (1.96)	1 (1.60)	0 (0.00)	0 (0.00)	0 (0.00)
20-29	1 (9.09)	8 (15.69)	9 (14.52)	*^a^* (*^a^*)	*^a^* (*^a^*)	6 (5.22)
30-39	1 (9.09)	6 (11.76)	7 (11.29)	3 (11.53)	5 (5.61)	8 (6.96)
40-49	3 (27.27)	12 (23.53)	15 (24.19)	7 (30.43)	13 (14.60)	20 (17.39)
50-59	4 (36.36)	8 (15.69)	12 (19.35)	7 (26.92)	17 (19.10)	24 (20.87)
60-69	0 (0.00)	12 (23.53)	12 (19.35)	5 (19.23)	17 (19.10)	22 (19.13)
70+	2 (18.18)	4 (7.84)	6 (9.68)	3 (11.53)	32 (38.96)	35 (30.43)
**Hormone hypersecretion** *^b^*						
None	4 (36.36)	18 (35.29)	22 (35.48)	Not available		
Cortisol	6 (54.54)	28 (54.90)	34 (54.84)			
Aldosterone	0 (0.00)	4 (7.84)	4 (6.45)			
Testosterone	1 (9.09)	10 (19.61)	11 (17.74)			
Estradiol	0 (0.00)	3 (5.88)	3 (4.84)			
Androstenedione	1 (9.09)	6 (11.76)	7 (11.29)			

Abbreviations: CCCR, Colorado Central Cancer Registry; CO, Colorado; CU, University of Colorado.

^a^Low values suppressed in accordance with CCCR's data use agreement.

^b^Some patients had tumors that secreted more than one hormone, resulting in percentages greater than 100%.

In the 115 patient cases diagnosed over 8 years identified in the state cancer registry, 56 (48.7%) were female, and 59 (51.3%) were male (see Table 1). Of interest, the CCCR data showed a higher proportion of diagnoses after age 70 (30.43%).

For comparison to ACC incidence, our Adrenal Cancer Center Program evaluated 115 patient cases of PPGL from Colorado who were diagnosed between 2016 and 2023. There was an age-adjusted state-wide average annual incidence estimate of 2.26 cases per million. Of these patient cases, 69 (60%) were female and 46 (40%) were male. The average age of PPGL diagnosis was 46.6 years, with the age distribution shown in Table 2. In addition, 53 (46.1%) of the primary tumors were adrenal, 16 (13.9%) were extra-adrenal, and 46 (40%) were located in the head and/or neck. Data for hormone secretion were available for 103 of these 115 patients, in whom it was detected in 60 (58.3%) of the tumors. Of these tumors, 47 produced normetanephrine/norepinephrine, 24 produced metanephrine/epinephrine, and 19 produced dopamine (see Table 2).

**Table 2.** Demographic variables of the 115 pheochromocytoma/paraganglioma patients in our University of Colorado cohort

Variable	PPGL patients
	N (%)
**Sex**	
Female	69 (60.00)
Male	46 (40.00)
**Age, years**	
<20	3 (2.61)
20-29	18 (15.65)
30-39	24 (20.87)
40-49	21 (18.26)
50-59	15 (13.04)
60-69	27 (23.48)
70+	7 (6.09)
**Tumor location**	
Adrenal	53 (46.09)
Extra-adrenal	16 (13.91)
Head and neck	46 (40.00)
**Secretion** *^a^*	
None	43 (41.70)
Normetanephrine/Norepinephrine	47 (45.65)
Metanephrine/Epinephrine	24 (23.32)
Dopamine	19 (18.48)

Abbreviation: PPGL, pheochromocytoma/paraganglioma.

^a^Secretion data collected for 103 patients. Some patients had tumors that secreted more than one hormone, resulting in percentages greater than 100%.

The geographic analysis of our ACC cohort showed that 11 of 62 (17.7%) patient cases resided in the Western part of the state, while the remaining 51 (82.3%) resided in the Eastern part of the state (Table 3). There were 0 to 3 cases per year of ACC from Western Colorado, and 1 to 8 cases per year from Eastern Colorado. However, Western Colorado is significantly less populated than Eastern Colorado. Thus, the age-adjusted 13-year cumulative incidence rates in Western and Eastern Colorado were quite different, with 17.69 and 8.95 cases per million, respectively. This equated to age-adjusted annual average incidence rates of 1.36 compared to 0.68 cases per million in Western compared to Eastern Colorado.

**Table 3.** The age-adjusted cumulative incidence of adrenocortical carcinoma (ACC) cases and the estimated average annual incidences, calculated for both our University of Colorado cohort (2010-2023) and for the Colorado State Cancer Registry ACC cohort (2012-2020)

Location	Total population	CU ACC cohort			CCCR ACC cohort		
		ACC cases	13-year cumulative incidence, cases per million	Average annual incidence, cases per million	ACC cases	8-year cumulative incidence, cases per million	Average annual incidence, cases per million
**Western Colorado**	585 682	11	17.69	1.36	26	11.92	1.49
**Eastern Colorado**	5 188 032	51	8.95	0.68	89	8.02	1.00
**Total in Colorado**	5 773 714	62	9.83	0.76	115	8.81	1.10

Abbreviations: CCCR, Colorado Central Cancer Registry; CU, University of Colorado.

In the CCCR ACC cohort, 26 out of 115 patient cases (22.6%) were in Western Colorado, with the remaining 89 (77.4%) from Eastern Colorado (see Table 3). Similar to our cohort, there was a geographic difference in ACC incidence. The age-adjusted 8-year cumulative incidence rate in Western Colorado was 11.92 cases per million, compared to 8.02 cases per million in Eastern Colorado. The average annual incidence rates of ACC were 1.49 per million in Western Colorado and 1 per million in Eastern Colorado, again demonstrating a divergent geographic distribution.

To compare with our ACC data, geographic analysis of PPGL cases showed an increased incidence in Eastern Colorado compared to Western Colorado (Table 4). The 8-year age-adjusted cumulative incidence was 10.83 cases per million in Western Colorado and 18.91 cases per million in Eastern Colorado, equating to annual average incidences of 1.35 cases per million and 2.36 cases per million, respectively.

**Table 4.** Age-adjusted cumulative incidence of pheochromocytoma/paraganglioma cases from 2016 to 2023 and the estimated average annual incidence calculated for our University of Colorado cohort

	Total population	PPGL cases	8-year cumulative incidence, cases per million	Average annual incidence, cases per million
**Western Colorado**	585 682	7	10.83	1.35
**Eastern Colorado**	5 188 032	108	18.91	2.36
**Total in Colorado**	5 773 714	115	18.07	2.26

Abbreviation: PPGL, pheochromocytoma/paraganglioma.

## Discussion

Based on the cases seen at our referral center, we estimated the yearly incidence of ACC to be 0.76 cases per million people, consistent with the most recent reports of US annual ACC incidence of 0.72 per million between 1973 and 2000 [2], as well as 1.02 per million between 1973 and 2014 [1]. Our data also align with a higher prevalence in women, with women in our cohort being 1.58 times more likely than men to develop ACC. Our CU cohort also confirmed prior publications of a peak in adult cases in patients aged 40 to 60 years [6]. About half of the patients’ tumors produced cortisol; the next most frequent hormone hypersecretion was testosterone, followed by androstenedione, aldosterone, and then estradiol (see Table 1). This pattern of hormonal secretion was consistent with other reports of the types of hormone secretion in ACC [7, 8].

The demographics of the Colorado State Cancer Registry ACC cohort, with a ratio of nearly 1:1 women to men, are in contrast to the published literature; with the prevalence of ACC occurring more commonly in women [9]. It also did not reflect the usual age distribution of ACC, as about 30% of the cohort was older than 70 years, with this increase evident in patient cases from Eastern Colorado. One possible explanation for this discrepancy is that there were errors in diagnosis or reporting of patients with ACC. ACC can be a difficult pathologic diagnosis to make [10] with evaluation of specialized criteria [11, 12] not followed at all centers. Similarly, there may be a lack of consistency in how different hospitals are reporting the data, such as direct reporting to the registry, pathology reports, or death certificates. Since PPGL is not reported in the Colorado state cancer registry at present, it is possible that some PPGLs have been falsely reported as other tumor types.

The results from both our patients and the state registry of ACC suggest that the cumulative incidence of ACC in Western Colorado is about 2 times higher than that in Eastern Colorado. To determine whether these results truly reflect of the characteristics of ACC incidence in Colorado or merely result from the way in which the analysis was conducted, we analyzed PPGL incidence from our center. Our data suggest an average annual PPGL incidence of 2.26 cases per million, lower than the reported international incidence of 6 cases per million [13, 14]. This may be attributed to PPGL being more common than ACC, and therefore, less likely to be referred to specialized adrenal tumor programs. These data suggested a higher PPGL incidence in Eastern Colorado, which would be expected, as our center is located in Eastern Colorado. This was not the case for our ACC cohort, however, which further supports our hypothesis of an increased ACC incidence in Western Colorado compared to the rest of the state.

There have been a few investigations into geographic distribution of ACC in the literature. An analysis of 448 ACC cases from the Texas State Cancer Registry did not identify differences in regional presentation [4]. However, another study, presented to date in abstract form, reported no increase in incidence but a shorter disease-free survival in ACC patients who reside in areas in Italy that have contaminated soil and water, suggesting differences in toxic exposures may play a role in the aggressiveness of the disease [5]. Interestingly, the Western half of Colorado is at a higher elevation, is more rural than urban, and is a site for mining and agriculture, which theoretically may predispose individuals to increased environmental toxins contributing to the geographic differences in ACC incidence.

We also investigated other differences between Western and Eastern Colorado that may contribute to the variable incidence of ACC. An analysis of socioeconomic data from the US Census Bureau showed that Western Colorado has a lower per capita income than Eastern Colorado, $42 866 compared to $47 865, respectively [15]. Furthermore, Western Colorado has a higher percentage of residents who are non-Hispanic White than Western Colorado, 75% compared to 64%, indicating less ethnic diversity in this region [16]. Lastly, data from the Colorado Rural Health Center classify only 1 of the 21 Western counties, and 16 out of the 43 Eastern counties, as urban [17], highlighting the differences in population density and potential differential exposures.

When considering environmental exposures that might differ between Western and Eastern Colorado, data from the Colorado Department of Public Health and Environmental demonstrate that areas around the Denver metropolitan area in Eastern Colorado had more days with above-maximum standard ozone concentrations [18], uranium levels in well water [19], and per-and polyfluoroalkyl substances (PFAS) levels in drinking water [20] than Western Colorado. These data would contradict our hypothesis for increased environmental risk in Western Colorado, and lead us to more strongly consider other factors. Although none of these data identify a clear cause of the geographical difference in ACC incidence, there may still be environmental differences relating to other water or soil contaminants potentially resulting from pesticide use or oil and mining activities, or there may be socioeconomic or behavioral differences to consider, such as physical activity level, nutrition, health care access, or occupational differences between those who live in these regions of Colorado.

Limitations to our study include that we were unable to analyze PPGL incidence in the state cancer registry, as it does not collect PPGL data. Another limitation is the lack of a longitudinal residential history of our ACC patients; only the zip code they resided in at the time of diagnosis was available, which might hinder investigation of potential environmental toxins contributing to the divergent incidence. Lastly, we are unsure of the reliability of the state cancer registry data, as ACC is a rare disease and may not be diagnosed properly at hospitals that do not have specialized adrenal tumor programs. However, since both our CU cohort and the state cancer registry data show increased ACC cases in Western Colorado, but our CU cohort does not show this pattern in PPGL, it is increasingly likely that there is a difference in geographic distribution of ACC cases in our state.

Despite these limitations, our study suggests geographical differences in the distribution of ACC from two registries in Colorado, our local database and the Colorado state cancer registry, which were not observed in our PPGL cases. There are differences in potential exposures between Western and Eastern Colorado, particularly those related to rural living, which are intriguing to hypothesize about as environmental risk for ACC. Ultimately, these results warrant further geographical analyses of ACC incidence in other states and countries, and further investigation into the potential role of environmental or other risk factors in the development of ACC.

## Data Availability

Some or all data sets generated during and/or analyzed during the present study are not publicly available but are available from the corresponding authors on reasonable request.

## Abbreviations

ACC: adrenocortical carcinoma
CCCR: Colorado Central Cancer Registry
CU: University of Colorado
PPGL: pheochromocytoma/paraganglioma
